# Morpho-functional evaluation of lung aeration as a marker of sickle-cell acute chest syndrome severity in the ICU: a prospective cohort study

**DOI:** 10.1186/s13613-019-0583-y

**Published:** 2019-09-30

**Authors:** Marc Garnier, El Mahdi Hafiani, Charlotte Arbelot, Clarisse Blayau, Vincent Labbe, Katia Stankovic-Stojanovic, François Lionnet, Francis Bonnet, Jean-Pierre Fulgencio, Muriel Fartoukh, Christophe Quesnel

**Affiliations:** 1 Département d’Anesthésie et Réanimation, APHP Hôpital Tenon, 4 rue de la Chine, 75020 Paris, France; 2 Service de Réanimation Médico-Chirurgicale, APHP Hôpital Tenon, Paris, France; 3 Université Pierre et Marie Curie Sorbonne Université, Paris, France; 4 Département d’Anesthésie et Réanimation, APHP Hôpital de la Pitié-Salpêtrière, Paris, France; 5 Service de Médecine Interne et Centre de Référence de la Drépanocytose, APHP Hôpital Tenon, Paris, France

**Keywords:** Acute chest syndrome, Sickle-cell disease, Acute lung injury, Lung ultrasound, Bedside spirometry

## Abstract

**Background:**

Acute chest syndrome (ACS) is the main cause of morbi-mortality in patients with sickle-cell disease in the intensive care unit (ICU). ACS definition encompasses many types of lung damage, making early detection of the most severe forms challenging. We aimed to describe ACS-related lung ultrasound (LU) patterns and determine LU performance to assess ACS outcome.

**Results:**

We performed a prospective cohort study including 56 ICU patients hospitalized for ACS in a tertiary university hospital (Paris, France). LU and bedside spirometry were performed at admission (D0) and after 48 h (D2). Complicated outcome was defined by the need for transfusion of ≥ 3 red blood cell units, mechanical ventilation, ICU length-of-stay > 5 days, or death. A severe loss of lung aeration was observed in all patients, predominantly in inferior lobes, and was associated with decreased vital capacity (22 [15–33]% of predicted). The LU Score was 24 [20–28] on D0 and 20 [15–24] on D2. Twenty-five percent of patients (14/56) had a complicated outcome. Neither oxygen supply, pain score, haemoglobin, LDH and bilirubin values at D0; nor their change at D2, differed regarding patient outcome. Conversely, LU re-aeration score and spirometry change at D2 improved significantly more in patients with a favourable outcome. A negative LU re-aeration score at D2 was an independent marker of severity of ACS in ICU.

**Conclusions:**

ACS is associated with severe loss of lung aeration, whose resolution is associated with favourable outcome. Serial bedside LU may accurately and early identify ACS patients at risk of complicated outcome.

## Background

Sickle-cell disease (SCD) is one of the most frequent haemoglobinopathies worldwide. Its overall prevalence is not well determined in developed countries, but its incidence is still increasing, reaching over 4500 newborns in France over the past 10 years [1]. Progress made in its management led to increased life expectancy in developed countries [2], however patients with SCD are still at risk of early mortality due to acute chest syndrome (ACS) [1]. Half of adult deaths arise in the intensive care unit (ICU), where ACS represents the main source of mortality [2–4]. ACS is also the most frequent vaso-occlusive event leading to ICU admission, and can lead to substantial morbidity [3]. Its typical presentation is a sudden onset of cough, dyspnoea, fever and rales, accompanied by new pulmonary infiltrate on chest radiography [5]. However, lung damage may vary from interstitial infiltrates to alveolar condensation, including atelectasis, pleural effusion, and in the worst case acute respiratory distress syndrome [6, 7]. Thus, a simple tool allowing estimating the initial extent of ACS-related lung injury and its early evolution is required to avoid delayed management and improve severe ACS patient care.

Bedside assessment of lung injury in severe ACS patients, and consequently of lung aeration, is challenging. Chest X-ray is now considered a very imperfect tool in ICU patients, showing low sensitivity and specificity particularly for the diagnosis of alveolar consolidation [8, 9]. CT-scan reported frequent large postero-lateral lung consolidations in severe ACS patients [9], but this exam cannot be used for daily bedside monitoring of ACS evolution. More recently, lung ultrasound (LU) has been reported as a reliable tool for diagnosis of lung condensation compared to CT-scan [8, 10]. Finally, a functional approach could be used to estimate the amount of aerated lung, notably by measuring the Inspiratory Vital Capacity (IVC) at bedside when patients performed incentive spirometry [11, 12].

Hence, we aimed to assess the reliability of a bedside morpho–functional evaluation of ACS-related lung aeration loss for early detection of SCD patients who experience a complicated course during their ICU stay.

## Methods

See Additional methods in Additional file 1.

This article follows the STROBE statement [13]. STROBE Checklist is available as an Additional file 2.

### Study population

All patients over 18 years suffering from severe ACS were prospectively included in this single-centre prospective observational study within the first 12 h of admission into the Tenon University Hospital ICU (Paris, France) between March 2013 and September 2014. ACS was defined as new pulmonary infiltrate on the chest X-ray and at least one of the following criteria: fever, cough, acute dyspnoea, thoracic pain, pulmonary crackles or tubal blowing sound [7, 11]. ACS severity was defined according to French recommendations (see Additional file 1: Methods) [11]. Patients re-admitted for a second severe ACS during the study period were not re-included. Patients with mixed thoracic disease, notably association of ACS with cardiogenic pulmonary oedema, were not included.

### Patient management

Inclusion did not modify patient care, conducted according to French recommendations [11]. Briefly, standard care included bed rest, oxygen therapy, respiratory physiotherapy, incentive spirometry, intravenous hydration, and folate supplementation. Pain was managed using an incremental protocol adapted from national recommendations [11] (Additional file 3: Figure S1). Indication of red blood cell transfusion followed recommendations [11] and was systematically validated jointly by a local SCD referent physician and the attending intensivist before transfusion. An empirical antimicrobial therapy combining cefotaxime and spiramycin was initiated when the patient had fever, and secondarily adapted according to the final microbiological results [11].

### Data collection and outcome definition

Clinical data were prospectively collected. Blood sampling for biological examinations was left to the discretion of the attending physicians. When available, biological data regarding blood gases, blood count, haemoglobin S dosage, liver function tests, and LDH concentrations were collected.

Complicated outcome was defined a priori according to previously published criteria, by occurrence of at least one of the following events: ≥ 3 red blood cell (RBC) units transfused during ICU stay [3, 9, 14]; invasive or non-invasive mechanical ventilation requirement [3, 5, 9]; ICU length-of-stay > 5 days [3, 14, 15]; or in-hospital death.

### Lung ultrasound

A first lung ultrasound (LU) examination was performed within the first 12 h of hospitalization in the ICU and recorded as video loops. Results were not provided to the attending physicians, ensuring that LU results did not affect patient management. A second LU examination was performed 48 h later for patients still hospitalized in the ICU by the same expert examiner (*EM*-*H*) who performed all the examinations of this study. LU was performed using a 4–6 MHz abdominal probe (Acuson^®^ CV70, Siemens, Germany), investigating the 12 lung regions as previously described [16, 17]. Then, two experts (*MG* and *CA*) independently examined the recorded LU video loops in random order. Each lung region was characterized by the worst ultrasound pattern observed, according to the four patterns previously described [16, 17] (normal aeration [N]; moderate loss of aeration [B1]; severe loss of aeration [B2]; and lung consolidation [C], see characteristic LU patterns in Additional file 4: Video). Then, LU Score (LUS) at D0 and D2, and LU Re-aeration Score between D0 and D2 were calculated as previously described [16, 17].

### Lung inspiratory vital capacity measurement

Lung inspiratory vital capacity (IVC) was measured during absence of pain (i.e., numeric pain scale ≤ 4) by the attending physiotherapist after 5 cycles of normal ventilation followed by a forced expiration, using a volumetric exerciser (Voldyne^®^ 2500, Teleflex^®^ Medical) within the first 12 h of hospitalization in the ICU and in a period of 4 h before or after LU examination. IVC was recorded as the mean value of 3 consecutive measurements, according to international guidelines [18].

### Statistical analysis

Data were expressed as median [25th–75th percentile]. Variables were analysed using non-parametric tests. Kaplan–Meier curves were plotted and a log rank test was performed to compare ICU and hospital length-of-stays. A multivariate analysis of the predictors of complicated outcome was performed using a logistic regression model. Multivariate analysis was performed using a model including parameters with *p* value ≤ 0.20 in univariate analysis. A final model adjusted for age, baseline Hb and LDH value at inclusion was used to test the independent value of dynamic parameters to assess patient outcome.

*p* < 0.05 was considered significant. Statistical analysis was performed with GraphPad Prism 6 (GraphPad Software, USA) and Statview 5.0 (SAS Institute Inc, USA).

## Results

### Patient characteristics

**Fig. 1:**
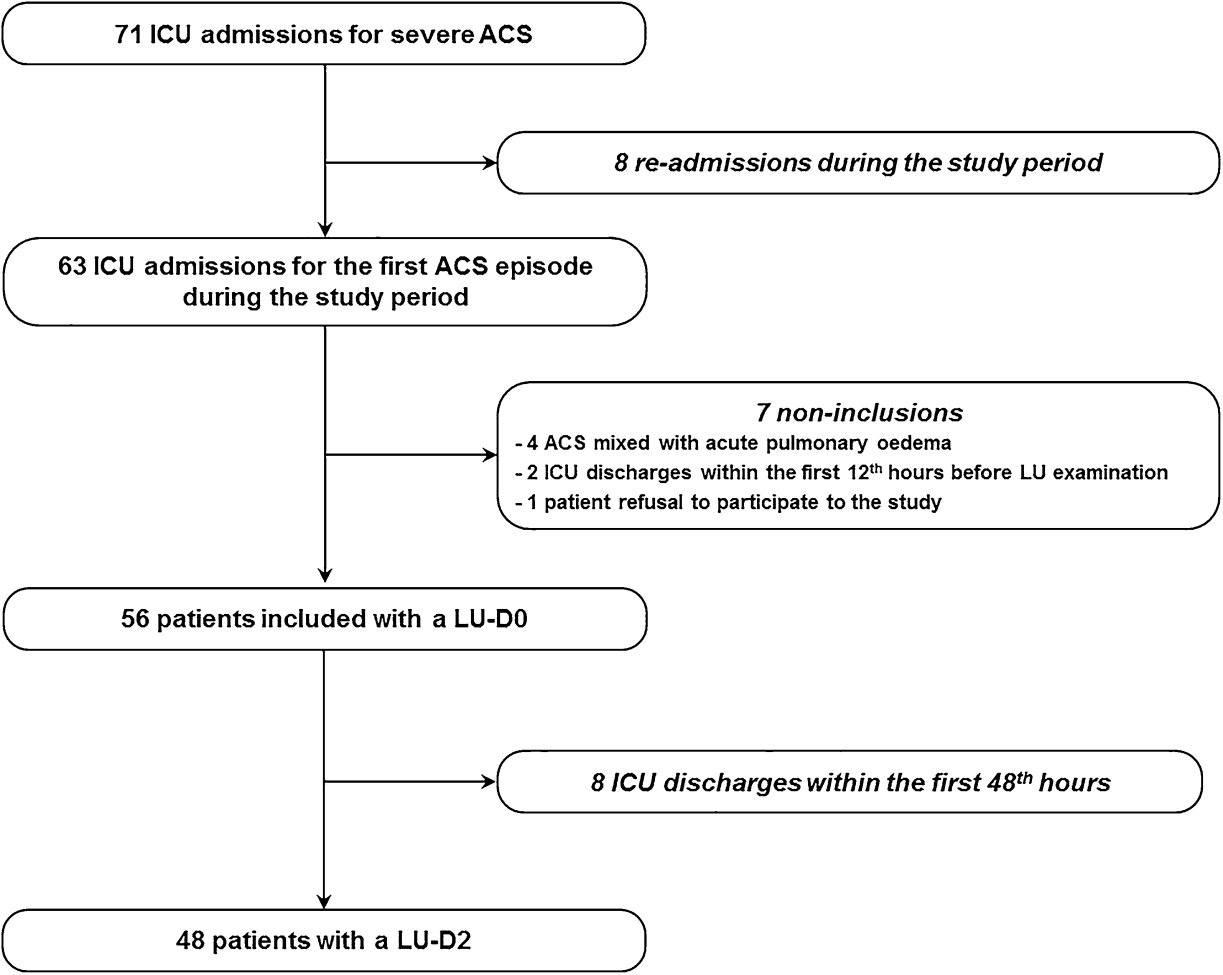
Flow diagram of the study

During the study period, 63 patients with severe ACS were referred to our unit, totalling 71 admissions. Among the 63 first episodes of ACS screened for eligibility, 56 were finally included (Fig. 1). Fourteen of the 56 patients (25%) had a complicated outcome, among whom 14 (100%) had an ICU length-of-stay > 5 days, 7 (50%) received ≥ 3 RBC units during their ICU stay, and 2 (14%) were mechanically ventilated. Baseline characteristics of patients with favourable and complicated outcomes were similar, except for a more frequent history of cholecystectomy in complicated patients (Table 1). Baseline severity of SCD, assessed by the severity score of Hebbel [19], modified by Lee et al. [20], was similar between the two groups (Table 1). Clinical and biological characteristics at inclusion were also similar between the two groups (Table 2).

**Table 1:** Baseline characteristics of patients

Patients characteristics	All patients (*n* = 56)	Patients with		*p*
		Favourable outcome	Complicated outcome	
		(*n* = 42)	(*n* = 14)	
Demographic data, median [IQR]				
Sex ratio (M/F)	22/34	17/25	5/9	0.75
Age (years)	26.4 [21.7–29.8]	27.4 [22.3–31.5]	22.1 [20.7–26.8]	0.06
BMI (kg/m^2^)	21.3 [19.8–23.6]	21.7 [19.8–23.4]	20.8 [20.2–24.7]	0.78
Cause of hospital admission, % (*n*)				
Vaso-occlusive crisis	48% (27)	52% (32)	43% (6)	0.34
Acute chest syndrome	46% (26)	45% (19)	50% (7)	
Others	6% (3)	2% (1)	7% (1)	
Type of hemoglobinopathy, % (*n*)				
SS	93% (52)	90% (38)	100% (14)	0.23
SC	7% (4)	10% (4)	–	
Baseline Hb (g/dL), median [IQR]	8.5 [7.5–9.5]	8.7 [7.8–9.5]	7.5 [7–9]	0.08
Number of vaso-occlusive crises/year, % (*n*)				
0	7% (4)	5% (2)	14% (2)	0.47
1–3	70% (39)	74% (31)	57% (8)	
4–6	21% (12)	19% (8)	29% (4)	
> 6	2% (1)	2% (1)	0% (0)	
Sickle cell disease-related complication, % (*n*)				
Previous acute chest syndrome	73% (41)	74% (31)	71% (10)	0.86
1–2	*34% (19)*	*31% (13)*	*43% (6)*	0.55
3–4	*25% (14)*	*26% (11)*	*21% (3)*	
> 4	*14% (8)*	*17% (7)*	*7% (1)*	
Cholecystectomy	35% (19)	26% (11)	57% (8)	0.03
Non-septic osteonecrosis	24% (14)	24% (10)	29% (4)	0.72
Renal injury	22% (12)	26% (11)	7% (1)	0.13
Retinopathy	20% (12)	21% (9)	21% (3)	1.00
Priapism	7% (4)	10% (4)	0% (0)	0.23
Others	15% (8)	14% (6)	14% (2)	1.00
Clinical severity score of Hebbel, median [IQR]	6 [3–9]	6 [3–9]	5.5 [3–8]	0.97

**Table 2:** Patient characteristics at inclusion

Patient characteristics	All patients (*n* = 56)	Patients with		*p*
		Favourable outcome	Complicated outcome	
		(*n* = 42)	(*n* = 14)	
Acute chest syndrome symptoms, % (*n*)				
Cough	70% (39)	69% (29)	71% (10)	0.17
Expectoration	50% (28)	52% (22)	43% (6)	0.54
Acute dyspnea	82% (46)	86% (36)	71% (10)	0.23
*Mild*	*35% (16/46)*	*33% (12/36)*	*40% (4/10)*	0.24
*Moderate*	*41% (19/46)*	*47% (17/36)*	*20% (2/10)*	
*Severe*	*24% (11/46)*	*19% (7/36)*	*40% (4/10)*	
Fever	84% (47)	83% (35)	86% (12)	0.83
Pulmonary rales	95% (53)	95% (40)	93% (13)	0.73
*Crackling sounds*	*75% (40/53)*	*78% (31/40)*	*69% (9/13)*	
Acute chest pain	93% (52)	93% (39)	93% (13)	1.00
*Numeric pain scale value*	*7 [6*–*9]*	*8 [6*–*9]*	*7 [5.3*–*8.5]*	0.39
Other pain	68% (38)	67% (28)	71% (10)	0.74
*Arms and/or legs*	*48% (27)*	*45% (19)*	*57% (8)*	0.36
*Back*	*23% (13)*	*24% (10)*	*21% (3)*	
*Abdomen*	*9% (5)*	*7% (3)*	*14% (2)*	
*Head and neck*	*5% (3)*	*5% (2)*	*7% (1)*	
Clinical characteristics, *median [IQR]*				
Heart rate (/min)	100 [87–111]	99 [86- 111]	102 [88–113]	0.73
Systolic blood pressure (mmHg)	130 [115–139]	130 [112–140]	125 [122–138]	0.93
Diastolic blood pressure (mmHg)	65 [59–75]	68 [59–75]	63 [61–69]	0.79
Respiratory rate (/min)	24 [20–29]	23 [20–29]	27 [23–31]	0.16
Inspiratory vital capacity (mL)	1000 [600–1250]	1000 [600–1300]	750 [500–1100]	0.12
SOFA score (points)	4 [3–4]	3 [3–4]	4 [3–4]	0.30
SAPS II (points)	15 [10.5–17]	14 [10–17]	16 [13–18]	0.32
Laboratory values, *median [IQR]*^a^				
Haemoglobin (g/dL)	7.5 [6.9–8.5]	7.5 [6.9–8.7]	7.4 [6.5–7.8]	0.18
*Haemoglobin S (*%*)*	81 [61–88]	80 [57–87]	83 [76–88]	0.21
Platelets (G/L)	327 [298–403]	329 [264–403]	325 [294–434]	0.76
Lactate dehydrogenase (UI/L)	544 [420–745]	503 [403–715]	613 [525–834]	0.09
Total bilirubin (µmol/L)	47 [33–79]	55 [38–79]	36 [29–44]	0.06
Aspartate aminotranferase (UI/L)	58 [36–73]	53 [38–69]	69 [35–77]	0.56
Alanine aminotransferase (UI/L)	33 [18–52]	32 [18- 49]	35 [18–51]	0.87
Blood gas results, *median [IQR]*^b^				
pH	7.39 [7.36–7.41]	7.39 [7.36–7.41]	7.38 [7.35–7.42]	0.71
PaO_2_ (mmHg)	81 [62–100]	80 [57–93]	96 [72–102]	0.18
*With delivered O*_*2*_ *flow (L/min)*	*3 [2*–*4.5]*	*3 [2*–*4.8]*	*3 [2*–*4]*	
PaO_2_/FiO_2_ ratio (mmHg)	259 [217–302]	254 [214–277]	300 [229–317]	0.19
PaCO_2_ (mmHg)	44 [40–48]	43 [41–48]	47 [39–49]	0.54
SaO_2_ (%)	95 [93–97.2]	95 [90–97]	97 [93.9–97.6]	0.27

*SOFA score* Sequential Organ Failure Assessment score, *SAPS II* Simplified Acute Physiologic Score II

^a^Biological data available for the 56 patients, except for haemoglobin S available for 41/56 patients (29 with a favourable outcome and 12 with a complicated outcome)

^b^Blood gas results available for 53 patients (39 with a favourable outcome and 14 with a complicated outcome)

### Clinico-biological data

Daily mean O_2_ flow during the 24 h after inclusion was 3.3 [2.3–4.7] L/min. Oxygen delivery decreased from D0 to D2 (Table 3). There was no difference between patients with and without complicated outcome.

**Table 3:** Data course and outcomes

Data course and outcomes	All patients (*n* = 56)	Patients with		*p*
		Favourable outcome	Complicated outcome	
		(*n* = 42)	(*n* = 14)	
Oxygen delivery (L/min), *median [IQR]*				
Mean O_2_ flow at D0	3.2 [2.3 to 4.5]	3.2 [2.3 to 4.3]	3.6 [2.6 to 4.9]	0.54
Mean O_2_ flow change at D2^a^	− 0.9 [− 2.4 to − 0.1]	− 1 [− 2.4 to − 0.2]	− 0.6 [− 1.3 to 0]	0.53
Numeric Pain Scale values, *median [IQR]*				
Mean NPS at D0	4 [2.4 to 6]	3.9 [2 to 6]	4.2 [3.9 to 6.3]	0.23
Mean NPS change at D2^a^	− 1.7 [− 3.5 to − 0.6]	− 1.7 [− 3.6 to − 0.8]	− 1.3 [− 3.4 to 0.1]	0.33
Respiratory rate (/min), *median [IQR]*				
Respiratory rate at D0	24 [20 to 29]	23 [20 to 29]	27 [23 to 31]	0.16
Respiratory rate change at D2^a^	− 5 [− 7 to − 1.5]	− 5 [− 7 to − 0.5]	− 4 [− 6 to − 2.8]	0.70
Laboratory values, *median [IQR]*				
Haemoglobin at D0 (g/dL)	7.5 [6.9 to 8.5]	7.5 [6.9 to 8.7]	7.4 [6.5 to 7.8]	0.18
Haemoglobin change at D2 (g/dL)^a^	+ 0.2 [− 0.6 to 1.5]	+ 0.1 [− 0.6 to 0.6]	+ 0.9 [− 0.1 to 1.5]	0.03
Platelets at D0 (G/L)	327 [298 to 403]	329 [264 to 403]	325 [294 to 434]	0.76
Platelets change at D2 (G/L)^a^	+ 18 [− 18 to 23]	+ 26 [0 to 74]	− 2 [− 34 to 38]	0.08
LDH at D0 (UI/L)	544 [420 to 745]	503 [403 to 715]	613 [525 to 834]	0.09
LDH change at D2 (UI/L)^a^	− 47 [− 153 to − 7]	− 58 [− 145 to − 18]	− 24 [− 205 to 90]	0.44
Bilirubin at D0 (µmol/L)	47 [33 to 79]	55 [38 to 79]	36 [29 to 44]	0.06
Bilirubin change at D2 (µmol/L)^a^	− 12 [− 29 to − 6]	− 15 [− 35 to − 6]	− 9 [− 12 to − 3]	0.10
SOFA score (points), *median [IQR]*				
SOFA score at D0	4 [3 to 4]	3 [3 to 4]	4 [3 to 4]	0.30
SOFA score change at D2^a^	− 1 [− 1 to 0]	− 1 [− 1 to − 1]	− 0.5 [− 1 to 0]	0.09
Lung ultrasound data, *median [IQR]*				
LU score at D0	24 [20 to 28]	25 [20 to 28]	22.5 [18 to 26]	0.17
LU score change at D2^a^	− 5 [− 11 to 1]	− 7 [− 12 to − 5]	+ 2 [− 2.5 to 5]	< 0.001
LU re-aeration score between D2 and D0^a^	5 [− 3 to 10.5]	7.5 [4.5 to 15]	− 3 [− 6.5 to 1.8]	< 0.001
Inspiratory vital capacity (mL), *median [IQR]*				
Inspiratory vital capacity at D0	1000 [600 to 1250]	1000 [600 to 1300]	750 [500 to 1100]	0.12
Inspiratory vital capacity change at D2^a^	+ 250 [25 to 500]	+ 500 [150 to 600]	+ 250 [0 to 250]	0.01
Empirical antimicrobial therapy, % (*n*)	98% (55)	98% (41)	100% (14)	0.56
Microbiologically documented pneumonia, % (*n*)	20% (11)	21% (9)	14% (2)	0.71
Transfusion, % (*n*) *or median [IQR]*				
Transfusion	57% (32)	45% (19)	93% (13)	0.005
Total number of RBC units	1 [0 to 2]	0 [0 to 2]	2 [2 to 3]	< 0.001
Mechanical ventilation, % (*n*)				
Non-invasive	2% (1)	0% (0)	7% (1)	0.02
Invasive	2% (1)	0% (0)	7% (1)	
Length-of-stay (days), *median [IQR]*				
ICU	5 [4 to 6]	4 [4 to 5]	7 [7 to 8]	–
Hospital	8 [6 to 11.3]	7 [5 to 9]	13 [10.3 to 14]	< 0.001
Death, % (*n*)	0% (0)	0% (0)	0% (0)	–

^a^Values for changes between D0 and D2 are reported for the 48 patients that were not discharged from the ICU before D2 (14 patients with a complicated and 34 with a favourable outcome)

Mean numeric pain scale during the 24 h following the inclusion was 4 [2.4˗6] (Table 3). Pain and corresponding morphine use significantly decreased from D0 to D2 (Additional file 3: Figure S2). There were no between-group differences. All patients were treated by at least 2 classes of analgesics, of whom 98% received acetaminophen, 93% morphine, 91% nefopam, 48% tramadol and 25% ketamine.

Haemoglobin values at D0 did not differ between patients with favourable and complicated outcomes. Haemoglobin values increased significantly more at D2 in patients with a complicated outcome but these patients were more transfused (Table 3). Neither platelets count, LDH and bilirubin values at D0, nor their change at D2, differed between patients with favourable and complicated outcomes (Table 3).

### Lung ultrasound

See details on LU patterns in Additional file 5: LU results.

**Fig. 2:**
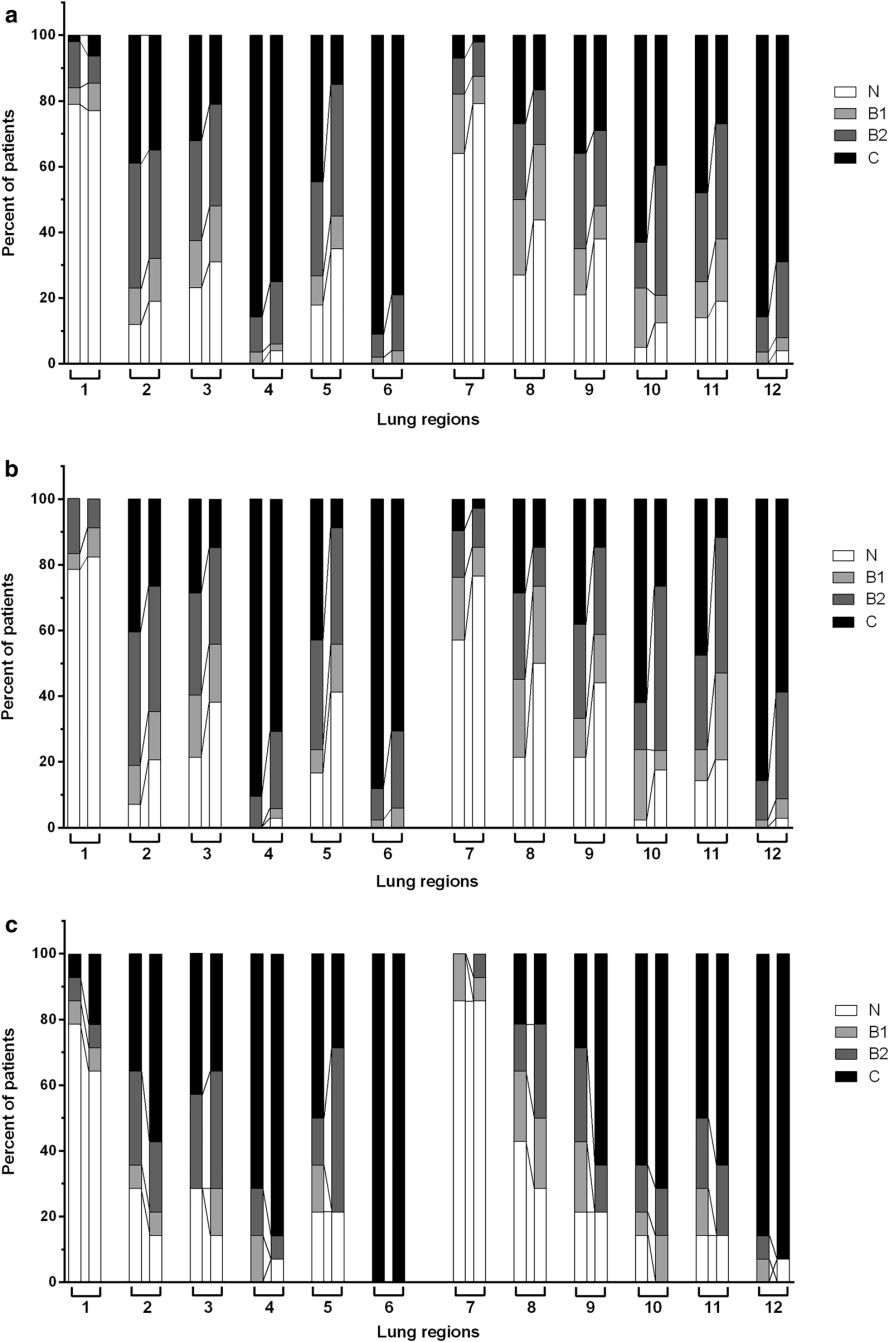
Distribution of the Lung Ultrasound (LU) patterns among the 12 lung regions for the whole cohort (**a**), and patients with a favourable (**b**) or a complicated (**c**) outcome, at inclusion *(left bar in each lung region)* and D2 *(right bar in each lung region)*. Lungs were divided in 12 regions: left and right antero-superior regions were numbered 1 and 7, respectively; antero-inferior regions 2 and 8; latero-superior regions 3 and 9; latero-inferior regions 4 and 10; postero-superior regions 5 and 11; and postero-inferior regions 6 and 12. The main LU pattern was large bilateral lung consolidations, predominantly distributed in dependent pulmonary areas (regions 4–6 for left lung and 10–12 for right lung). “*N”: normal aeration; “B1”: moderate loss of aeration; “B2”: severe loss of aeration; “C”: lung consolidation*

At inclusion, a loss of aeration was found in all lung regions for the majority of patients, except for antero-superior regions, without any difference according to patient outcome (Fig. 2). Forty-three patients (80%) had bilateral postero-inferior lung consolidations. The LUS was 24 [20–28], without any difference according to patient outcome (Additional file 3: Figure S3A and Table 3).

At D2, LU patterns showed a moderate regression of aeration loss, with lung consolidation still reaching 79% and 69% of patients for left and right postero-inferior regions, respectively (Fig. 2). The LUS decreased from 24 [20–28] to 20 [15–24] between D0 and D2 (*p* < 0.001), with more improvement in patients with a favourable outcome (Additional file 3: Figure S3A). The overall LU re-aeration score between D0 and D2 was 5 [− 3 to 9.5]. Patients with a complicated outcome had a significantly lower re-aeration score than their counterparts (− 3 [− 6.5 to 1.8] vs. 7.5 [4.5–15], *p* < 0.001) (Additional file 3: Figure S3B and Table 3**)**.

### Lung inspiratory vital capacity

IVC was dramatically impaired at D0 (1000 [600–1250] mL, corresponding to 24 [16–35]% of the predicted forced vital capacity). Overall IVC significantly increased between D0 and D2 (1250 [1000–1690] mL, *p* = 0.05 vs. D0). Patients with a complicated outcome had a significantly lower IVC increase at D2 than their counterparts (+ 250 [0–250] vs. + 500 [150–600] mL, *p* = 0.003; Table 3). IVC change between D0 and D2 was correlated with the LU re-aeration score (*r* = 0.66—*p* < 0.0001—Additional file 3: Figure S4).

### Outcomes

#### ICU and hospital length-of-stays

**Fig. 3:**
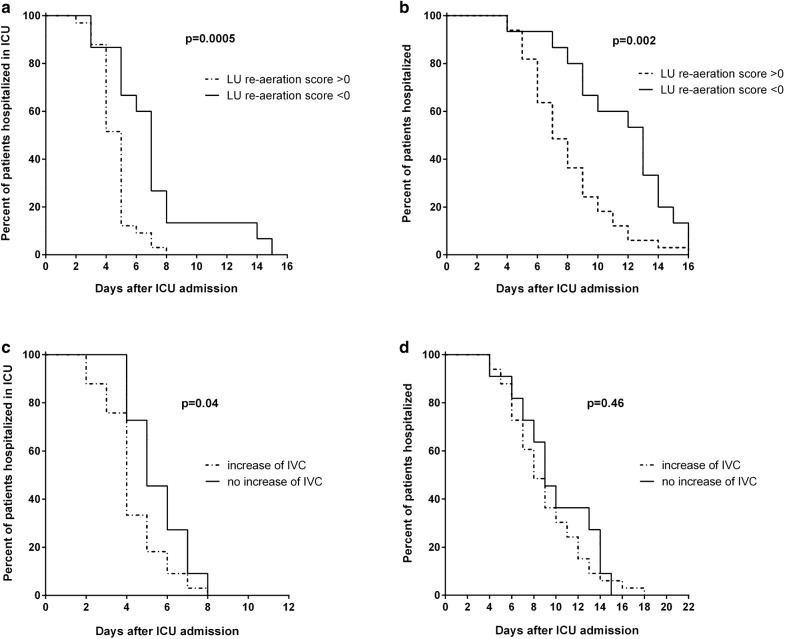
Length-of-stay in the intensive care unit (ICU) and in the hospital according to the lung aeration assessed by the lung ultrasound (LU) re-aeration score or the inspiratory vital capacity (IVC). Patients with a negative LU re-aeration score had longer stays in ICU (**a**) and in hospital (**b**) than patients with a positive LU re-aeration score. Patients with an increased IVC at D2 had a slightly shorter ICU stay than patients without IVC increase (**c**), while there were no differences between groups in hospital length-of-stay (**d**)

Overall, ICU and hospital length-of-stays were 5 [4–5.3] and 8 [6–11.3] days after inclusion, respectively. Compared to patients with a favourable outcome, length-of-stay of patients with a complicated outcome, by definition longer in ICU, was also significantly prolonged for the total hospital stay (Table 3). Patients with a positive LU re-aeration score had shorter ICU and hospital length-of-stays than patients with a negative re-aeration score (Fig. 3a, b). Patients with an increase of IVC at D2 had shorter ICU length-of-stay than patients without IVC increase, whereas there was no difference in hospital length-of-stay (Fig. 3c, d).

#### Transfusion

Thirty-two patients (57%) were transfused with 2 [2] RBC units, among whom 13 had a combined phlebotomy. Among the 7 patients with complicated outcome who were transfused with ≥ 3 RBC units, the third RBC unit was transfused 2 [2–3] days after inclusion. Patients with a positive LU re-aeration score had significantly fewer transfusion episodes (Additional file 3: Figure S5A), and received fewer RBC units (1 [0–2] vs. 2 [1–3] units, *p* = 0.003) than patients with a negative LU re-aeration score. Conversely, there was no difference between patients with and without IVC increase in the number of transfusion episodes and RBC units transfused (1 [0–2] vs. 2 [0–3] units, *p* = 0.37) (Additional file 3: Figure S5B).

#### Mechanical ventilation and death

Finally, 2 patients were mechanically ventilated (1 non-invasively at day 5 post-inclusion and 1 invasively at day 9 post-inclusion), both with a negative LU re-aeration score but an increased IVC at D2. No deaths were observed.

### Parameters associated with outcome

**Fig. 4:**
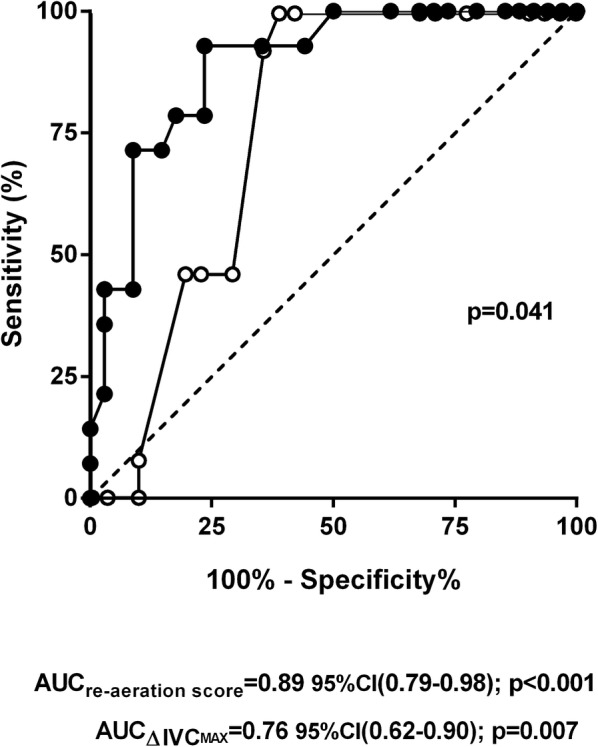
Receiver operating characteristic (ROC) curves for the lung ultrasound (LU) re-aeration score (black symbols) and the inspiratory vital capacity (IVC) change (white symbols) for the diagnosis of acute chest syndrome (ACS) severity. If both the LU re-aeration score and IVC change had good accuracy for diagnosing ACS severity, the area under the ROC curve (AUC) for the LU re-aeration score was significantly better than that of IVC

In univariate analysis, the only parameters significantly associated with the composite outcome criterion were LU re-aeration score and IVC change at D2 (Additional file 6: Table S1). After adjustment for age, baseline Hb and LDH value at inclusion, LU re-aeration score and IVC change at D2 remained independently associated with outcome (OR 0.62 [0.43–0.89] per point and 0.66 [0.46–0.95] per 100 mL, respectively) (Additional file 6: Table S1). The best area under the ROC curve associated with complicated outcome was obtained for the LU re-aeration score (0.87 [0.79–0.98] vs. 0.76 [0.62–0.90] for IVC change, *p* = 0.041) (Fig. 4). A negative LU re-aeration score between D0 and D2 (i.e., cut-off value of 0) had 71.4% sensitivity, 91.2% specificity and a positive likelihood ratio of 8 to predict complicated outcome.

## Discussion

Our main results may be summarized as follows: (1) severe ACS patients had a considerable morphological and functional loss of lung aeration and (2) early variation of lung aeration loss estimated by a bedside morpho–functional approach is independently associated with complicated outcome.

In our study, the main ACS morphological pattern was large bilateral lung consolidations, predominantly distributed in dependent pulmonary areas. Our results are concordant with previous CT-scan reports [9, 14, 21] and with the only other study that used LU in ACS patients admitted to the ICU [22]. Lungs of ACS patients showed a dramatic loss of aeration leading to severe impairment of vital capacity and high initial LUS. Distribution of ACS lung consolidation does not evocate classical bacterial pneumonia, in which no zonal predilection is usually reported [16, 23]. Thus, non-infectious mechanisms may also act in ACS, such as inflammatory oedema, diaphragmatic course limitation, and lower lobe collapse, notably due to the pressure exerted by a dilated right heart [9]. However, ACS patient tolerance of this severe acute lung injury is startlingly good, requiring mechanical ventilation in less than 5% of cases in our cohort, as in other recent reports [9, 24]. The discrepancy between the severity of morphologic and functional lung damages and the moderate hypoxemia observed in these patients may contribute to the low incidence of mechanical ventilation. Such a discrepancy, in our study as in others [9, 22], may be, at least in part, explained by enhanced microvascular response to hypoxia [25, 26] leading to increased acute hypoxic pulmonary vasoconstriction. In addition, the functional shunt in the collapsed lung may be further reduced by sickle red blood cells entrapment in these most hypoxic regions, leading to a large redistribution of blood flow toward the upper lobes. This might contribute to explain the existence of an association between the morpho–functional evaluation of lung aeration loss and the outcome, and the absence of such an association for oxygenation parameters.

Early detection of ACS patients who will experience a complicated outcome is a major concern, since it would allow physicians to modify their management accordingly. To date, a simple and easily accepted diagnostic tool of ACS severity is lacking for severe patients admitted to the ICU. None of the specific tools associated with worsening evolution in ACS patients hospitalized in the wards (such as LDH concentration) [27] or in SCD patients referred to the ICU for mixed causes (such as changes in haemoglobin level or respiratory rate) [3], were associated with complicated outcome in our study. In addition, platelets count that has been reported to be predictive of rapidly progressive ACS [28], and its change between D0 and D2, were similar between patients with favourable and complicated outcomes. Eventually, general ICU prognostic tools such as SAPSII or SOFA scores at inclusion were not associated with complicated outcome, nor did SOFA change between D0 and D2. On the other hand, the early change of lung aeration loss appeared as a good severity parameter, morphologically estimated by LU and functionally estimated by bedside spirometry. If both evaluate lung aeration, they did not strictly evolve in a parallel manner, suggesting that a dual morphological and functional evaluation might be interesting. Indeed, several confounders other than lung aeration can influence vital capacity measurement, such as chest pain, patient tiredness, or even the method used to perform spirometry as reported in out-patients [29, 30]. Another limit of bedside spirometry to predict outcome is that the difference between the increase in vital capacity among patients with a favourable and a complicated outcome is weak, often inferior to the intra-individual variations that were observed within the repeated measures performed on each patient during the study. Thus, using only bedside spirometry as a predictive tool may be a source of error.

LU examination appears as an interesting complementary tool. In our study, LU showed the best area under the ROC curve associated with complicated outcome. LU allows for visual assessment of lung aeration, also providing the regional patterns of lung injury. LU is also useful in diagnosing pleural effusion [8, 31], and providing information on diaphragm function [32–34]. Some possible inconsistency of the lung ultrasound score (LUS) and a perfectible points allocation scale have been pointed out [35]. Indeed, “0 point” in an individual lung region does not necessarily reflect normal lung aeration and “3 points” complete loss of lung aeration. However, the LUS is a simple tool to provide a numerical quotation of the lung aeration that offers the advantages of being quickly calculated and easily understood by all LU practitioners. In addition, its validity is reinforced by the highly reproducible rating of lung aeration between examiners, in our study as in others [8], meaning that a LUS improvement most likely corresponds to a regression of the lung aeration loss. Finally, LU allows for bedside assessment of lung aeration without exposing patients to X-ray. This is an obvious advantage given the recurrence of ACS episodes during the lifespan of SCD patients, who receive an important dose of radiations among their life with current diagnostic strategies, among which an important part could be avoided implementing LU.

LU presents some limitations, among which availability of the technique and experience of physicians are probably the two most recurrent ones. The usefulness of LU in critically ill patients is now clearly demonstrated [31], leading to considerable improvement in availability of the technique. The development of an easy-to-use single-probe portable device mainly dedicated to LU could be a solution to democratize its use definitively. In addition, if training of ICU physicians has recently significantly increased, practicing in good quality training centres remains essential to improve learning and reinforce LU diagnostic performances. In these conditions, the learning curve of LU is short [36, 37], which could allow any physician involved in the management of SCD patients in the ICU, in the emergency room and even in the wards to quickly acquire the skills required to follow the ACS-induced lung aeration loss [38]. Then, it could be hypothesized that LU may be made in first line, and if possible replace chest X-ray for ACS diagnosis and follow-up in the near future, notably as chest X-ray performed in ICU lacks sensitivity and specificity in particular in ACS patients [9].

Our study presents some limitations. Conversely to previous results reported by Razazi et al. [22], initial LU seems not sufficient to determine severity and only dynamic parameters were informative in our study. This difference may be explained by the use of different scoring systems to transform LU images into numerical results between the two studies. This result may also be due to a lack of power related to the number of patients assessed with LU at D0 in our study. Eventually, this result may be explained by a difference in severity between the populations included in the two studies, in particular as the transfusion rate was 82% in the study of Razazi et al. [22] and 57% in the present one. However, all the other clinical and biological parameters reflecting the severity of an ACS are similar between the two cohorts. This rather suggests that our transfusion policy, based on a systematic joint assessment of the indication of the transfusion between the intensivist and the SCD referring physician and on the evaluation of the clinical course over the first 6 h in the ICU before transfusing, may be more restrictive than that of other centres. Thus, it could be hypothesized that the discrepancy between LU results at D0 and patient’s severity may be explained by the contribution of a chronic loss of aeration existing at steady state. Indeed, it was recently reported that 3 among 14 (21%) patients with SCD presenting to the emergency department for pain crisis and who did not developed ACS had however echographic lung consolidations [39]. These results suggest that lung aeration loss could exist chronically in SCD patients at steady state and/or occur in the event of an acute non-thoracic SCD-related event. Thus, a consolidation threshold associated with severity may be an interesting diagnostic tool but needs to be specified in larger studies. In this context, the early evolution of LUS, comparing data to those obtained in the same patient shortly before, seems a more reliable strategy to diagnose ACS severity. This questions the clinical relevance of such a two-point strategy and its real impact on patient care. In our study the evolution of lung aeration during the first 2 days was strongly associated with outcome events occurring beyond this time point. This requires further determination of the optimal timing for lung aeration re-assessment.

## Conclusion

Patients with severe ACS have a dramatic loss of lung aeration. Serial bedside spirometry and LU examination accurately assess the ACS-related lung aeration loss and allow for early identification of patients who will experience a complicated outcome. A negative LU re-aeration score is the best independent parameter associated with a complicated course among all clinical and complementary examination parameters. Together, our results suggest that early evolution of lung aeration loss could be a reliable criterion to define ACS severity.

## Supplementary information

**Additional file 1.** The additional method file provides additional details on methods used in the study.

**Additional file 2.** The additional file provides the STROBE checklist related to the content of the manuscript.

**Additional file 3.** The additional figures file provides 5 additional figures.

**Additional file 4.** The additional video file provides LU video loops of the 4 characteristic LU patterns.

**Additional file 5.** The additional results file provides additional results on lung ultrasound examination.

**Additional file 6.** The additional table file provides the results of the multivariate analysis of parameters associated with complicated outcome.

## Data Availability

The data sets used and analysed during the current study are available from the corresponding author on reasonable request.

## Abbreviations

ACS: acute chest syndrome
CT-scan: computed tomography scan
ICU: intensive care unit
IVC: inspiratory vital capacity
LU: lung ultrasound
LUS: lung ultrasound score
LUS-D0: lung ultrasound score at inclusion (D0)
LUS-D2: lung ultrasound score 48 h after inclusion (D2)
RBC: red blood cell
SCD: sickle-cell disease
