# Supplementary material for: Morpho-functional evaluation of lung aeration as a marker of sickle-cell acute chest syndrome severity in the ICU: a prospective cohort study
Source: Ann Intensive Care. 2019 Sep 30;9:109. doi: 10.1186/s13613-019-0583-y (PMC6766460; doi:10.1186/s13613-019-0583-y)
Supplement: Supplementary file 1 — Additional file 1. The additional method file provides additional details on methods used in the study. [file 13613_2019_583_MOESM1_ESM.docx]

**Morpho-functional Evaluation of Lung Aeration as a Marker of Sickle-Cell Acute Chest Syndrome severity in the ICU: a Prospective Cohort Study**

**Additional Methods**

**Study population and ethical considerations**

This single-centre prospective observational study was approved by the Institutional Review Board (Comité de Protection des Personnes (CPP) “Ile-de-France VI”, Paris, France, #07-02-2013), and the French national commission on digital storage of personal data (CNIL, n°1643704). Information was given directly to patients who accepted data and LU videos collection.

All consecutive patients aged over 18 years suffering from severe ACS were included within the first 12 hours following ICU admission in Tenon University Hospital (Paris, France) for 18 months. ACS was defined as new pulmonary infiltrate on the chest X-ray consistent with alveolar consolidation involving at least one complete lung segment, and at least one of the following criteria: fever, cough, acute dyspnea, thoracic pain, pulmonary crackles or tubal blowing sound [1, 2]. Severe ACS was defined by the presence of at least one of the following criteria [1]: respiratory rate >30/min or <10/min, shallow breathing, inability to speak, consciousness disorders, heart rate >120/min, association with another organ failure (i.e. acute renal insufficiency, cardiac insufficiency, etc.), hypoxemia with PaO_2_ <60 mmHg on room air on blood gases, respiratory acidosis with pH <7.35 or PaCO_2_ > 50 mmHg, > 2 lobes with lung injury or bilateral lung injury on chest ray, need for oxygen > 4 L/min to maintain SpO_2_ > 98% [2]. In the presence of at least 1 severity criterion, patients were systematically evaluated jointly by the attending SCD physician and an intensivist. Then, patients were transferred to the ICU in the presence of ≥2 severity criteria or 1 severity criterion associated with severe hypoxemia.

Patients re-admitted in ICU for a second ACS episode during the study period were not re-included. Patients with mixed thoracic diseases, in particular association of ACS with cardiogenic pulmonary oedema, were not included.

**Patient Management**

Inclusion in this protocol did not modify patient care, conducted according to French recommendations [2]. Standard care included bed rest until chest pain and/or extra-thoracic pain were sufficiently relieved to go in the chair. Facial oxygen therapy was initiated and O_2_ flow was set in order to maintain patients’ SpO2 above 95%. Respiratory physiotherapy was performed twice a day by 2 trained physiotherapists dedicated to the ICU, by focusing on the less painful moments. Incentive spirometry was supervised by physiotherapists and systematically performed at least twice a day. Patients were systematically supplemented with folate intravenously and then orally as soon as normal oral uptake resumed.

Pain was managed using an incremental protocol adapted from national recommendations [2] (***Additional*** ***Figure 1*** *in the online data supplement*). Except in case of contraindication, acetaminophen and nefopam were systematically used. Then, in case of severe pain insufficiently relieved, morphine was administered using Patient-Controlled Analgesia (PCA) with bolus starting at 1 mg without continuous infusion, and then increased as needed. When morphine boluses reached 3 mg, a 1-mg/h continuous intravenous morphine infusion was added to the PCA. If the continuous morphine infusion was still insufficient to relieve patients’ pain, the attending physicians prescribed a rescue analgesic treatment using ketamine. Tramadol could be used before morphine requirement or in relay after PCA withdrawal. When an anxiety component contributed to pain, hydroxyzine, clorazepate or cyamemazine could be used.

Indication of red blood cell transfusion followed the national recommendations [2]. Blood transfusion was indicated in the presence of at least one ACS severity criterion and: a haemoglobin value <6 g/dL with clinical impact, or no ACS improvement after 72h of management, or patient in the peripartum or postoperative phase. Blood transfusion was administered alone when anaemia was pronounced with haemoglobin values below baseline haemoglobin levels, and combined with phlebotomy when haemoglobin values were above baseline haemoglobin levels while clinical evolution worsened. Indication of transfusion was systematically jointly by a local SCD referent physician and the attending intensivist before transfusion.

An empirical antimicrobial therapy combining cefotaxime and spiramycin was initiated when the patient had fever, and secondarily adapted considering the definite microbiological results [2].

**Data collection and outcome definition**

General demographic data, SCD medical history, transfusions requirement and ICU and hospital lengths of stay were collected. Blood sampling for biological laboratory examinations was left at the discretion of the attending physicians. When available, biological data regarding blood gases, blood count, haemoglobin S dosage, bilirubin and liver function tests, and LDH concentrations were prospectively collected. Finally, daily mean and maximal values of delivered oxygen flow and numeric pain scale, and analgesics consumption were collected.

Complicated outcome was defined *a priori* according to previously published criteria, by occurrence of at least one of the following events: ≥3 red blood cell (RBC) units transfused during ICU stay [3-5]; invasive or non-invasive mechanical ventilation requirement [3, 4, 6]; ICU length-of-stay >5 days [3, 5, 7]; or in-hospital death.

**Lung Ultrasound**

A first Lung Ultrasound (LU) examination was performed within the first 12h of hospitalization in the ICU and recorded as video loops. Results were not provided to the attending physicians, ensuring that LU results do not affect management of patients. A second LU examination was performed 48h later for patients still hospitalized in the ICU by the same expert examiner (*MH*) who performed all the examinations of this study. LU was performed using a 4-6 MHz probe (Acuson^®^ CV70, Siemens, Germany), investigating the 12 lung regions as previously described [8, 9]. Then, two experts (*MG & CA*) independently examined the recorded LU video loops in a random order, each expert being blinded of the rating made by the other. Each lung region was characterized by the worst ultrasound pattern observed, according to the four ultrasound patterns previously described [8, 9] (i.e. normal aeration [N]; moderate loss of aeration [B1]; severe loss of aeration [B2]; and lung consolidation [C]). Discordances between the two ratings were individually discussed to consensually determine the definitive value. Then, LU Score (LUS, calculated as the sum of the individual score of the 12 lung regions, as follows: N=0 point; B1=1 point; B2=2 points; C=3 points) was calculated for LU-D0 and LU-D2. Then, the LU re-aeration score between D2 and D0 was calculated as the sum of the individual score of LU pattern change in the 12 lung regions, as previously described [8, 9] (*see table just above*). Finally, the presence of static and dynamic bronchogram, and the presence of pleural effusion were recorded.

***Lung re-aeration score calculation, from [8]***

| - 5 points | -3 points | -1 point | 0 point | +1 point | +3 points | +5 points |
| --- | --- | --- | --- | --- | --- | --- |
| N > C | N > B2  B1 > C | N > B1  B1 > B2  B2 > C | No change | B1 > N  B2 > B1  C > B2 | B2 > N  C > B1 | C > N |

**Lung Inspiratory Vital Capacity measurement**

Lung maximal Inspiratory Vital Capacity (IVC) was measured during painless moments (*i.e.* Numeric Pain Scale ≤4) by the attending physiotherapist after 5 cycles of normal ventilation followed by a forced expiration, using a volumetric exerciser (Voldyne^®^ 2500, Teleflex^®^ Medical) within the first 12h of hospitalization in the ICU and in a period of 4h before or after LU examination. IVC was recorded as the mean value of 3 IVC measurements. Predicted Vital Capacity was calculated according to the most recent recommendations through the 2012 Global Lung function Initiative equations [10], using the GLI-2012 Excel Individual Calculator (available at: www.ers-education.org/guidelines/global-lung-function-initiative/spirometry-tools/excel-individual-calculator.aspx).

**Statistical analysis**

Data were expressed as median and interquartile [25^th^-75^th^ percentile]. Demographic and clinical data were analysed using the chi-square test or the Fisher exact test for categorical parameters and the non-parametric Mann-Whitney *U* test for continuous variables. Within-group changes were analysed using the non-parametric paired Wilcoxon test. Kruskall-Wallis test with Dunn’s correction was used to compare more than two groups of continuous variables. Kaplan-Meier curves were plotted and a log rank test was performed to compare the duration of hospitalization between patients with a loss or a gain of lung aeration between D0 and D2. A multivariate analysis of the predictors of complicated outcome was performed using a logistic regression model. First, univariate analysis of the most relevant SCD baseline characteristics and clinical and paraclinical data at inclusion was performed. Then, a multivariate analysis was performed using a model including parameters with *p* value ≤ 0.20 in univariate analysis. A final model adjusted on age, baseline Hb and LDH value at inclusion (reflecting SCD and acute haemolysis severity) was used to test the independent value of dynamic parameters to assess patient outcome. The area under the receiver operating characteristics (ROC) curves for the dynamic parameters independently associated with complicated outcome in the multivariate analysis were calculated and compared.

A P value <0.05 was considered as significant. Statistical analysis was performed with GraphPad Prism 6 (GraphPad Software, San Diego, CA, USA) and Statview 5.0 (SAS Institute Inc, Cary, NC, USA).

**References**

1. Vichinsky EP, Neumayr LD, Earles AN, Williams R, Lennette ET, Dean D, Nickerson B, Orringer E, McKie V, Bellevue R, Daeschner C, Manci EA. Causes and outcomes of the acute chest syndrome in sickle cell disease. National Acute Chest Syndrome Study Group. *N Engl J Med* 2000;342:1855–1865.

2. Habibi A, Arlet J-B, Stankovic K, Gellen-Dautremer J, Ribeil J-A, Bartolucci P, Lionnet F, centre de référence maladies rares « syndromes drépanocytaires majeurs ». [French guidelines for the management of adult sickle cell disease: 2015 update]. *Rev Médecine Interne Fondée Par Société Natl Francaise Médecine Interne* 2015;36:5S3-5S84.

3. Cecchini J, Lionnet F, Djibré M, Parrot A, Stojanovic KS, Girot R, Fartoukh M. Outcomes of adult patients with sickle cell disease admitted to the ICU: a case series*. *Crit Care Med* 2014;42:1629–1639. .

4. Mekontso Dessap A, Deux J-F, Habibi A, Abidi N, Godeau B, Adnot S, Brun-Buisson C, Rahmouni A, Galacteros F, Maitre B. Lung imaging during acute chest syndrome in sickle cell disease: computed tomography patterns and diagnostic accuracy of bedside chest radiograph. *Thorax* 2014;69:144–151.

5. de Prost N, Sasanelli M, Deux J-F, Habibi A, Razazi K, Galactéros F, Meignan M, Maître B, Brun-Buisson C, Itti E, Mekontso Dessap A. Positron Emission Tomography With 18F-Fluorodeoxyglucose in Patients With Sickle Cell Acute Chest Syndrome. *Medicine (Baltimore)* 2015;94:e821.

6. Vichinsky EP, Neumayr LD, Earles AN, Williams R, Lennette ET, Dean D, et al. Causes and outcomes of the acute chest syndrome in sickle cell disease. National Acute Chest Syndrome Study Group. N Engl J Med. 2000;342:1855–65.

7. Tawfic QA, Kausalya R, Al-Sajee D, Burad J, Mohammed AK, Narayanan A. Adult Sickle Cell Disease: A Five-year Experience of Intensive Care Management in a University Hospital in Oman. *Sultan Qaboos Univ Med J* 2012;12:177–183.

8. Bouhemad B, Liu Z-H, Arbelot C, Zhang M, Ferarri F, Le-Guen M, Girard M, Lu Q, Rouby J-J. Ultrasound assessment of antibiotic-induced pulmonary reaeration in ventilator-associated pneumonia. *Crit Care Med* 2010;38:84–92.

9. Bouhemad B, Brisson H, Le-Guen M, Arbelot C, Lu Q, Rouby J-J. Bedside ultrasound assessment of positive end-expiratory pressure-induced lung recruitment. *Am J Respir Crit Care Med* 2011;183:341–347.

10. Quanjer PH, Stanojevic S, Cole TJ, Baur X, Hall GL, Culver BH, Enright PL, Hankinson JL, Ip MSM, Zheng J, Stocks J, ERS Global Lung Function Initiative. Multi-ethnic reference values for spirometry for the 3-95-yr age range: the global lung function 2012 equations. *Eur Respir J* 2012;40:1324–1343.
