# Supplementary material for: Morpho-functional evaluation of lung aeration as a marker of sickle-cell acute chest syndrome severity in the ICU: a prospective cohort study
Source: Ann Intensive Care. 2019 Sep 30;9:109. doi: 10.1186/s13613-019-0583-y (PMC6766460; doi:10.1186/s13613-019-0583-y)
Supplement: Supplementary file 3 — Additional file 3. The additional figures file provides 5 additional figures. [file 13613_2019_583_MOESM3_ESM.docx]

**Morpho-functional Evaluation of Lung Aeration as a Marker of Sickle-Cell Acute Chest Syndrome severity in the ICU: a Prospective Cohort Study**

**Additional Figures**

**
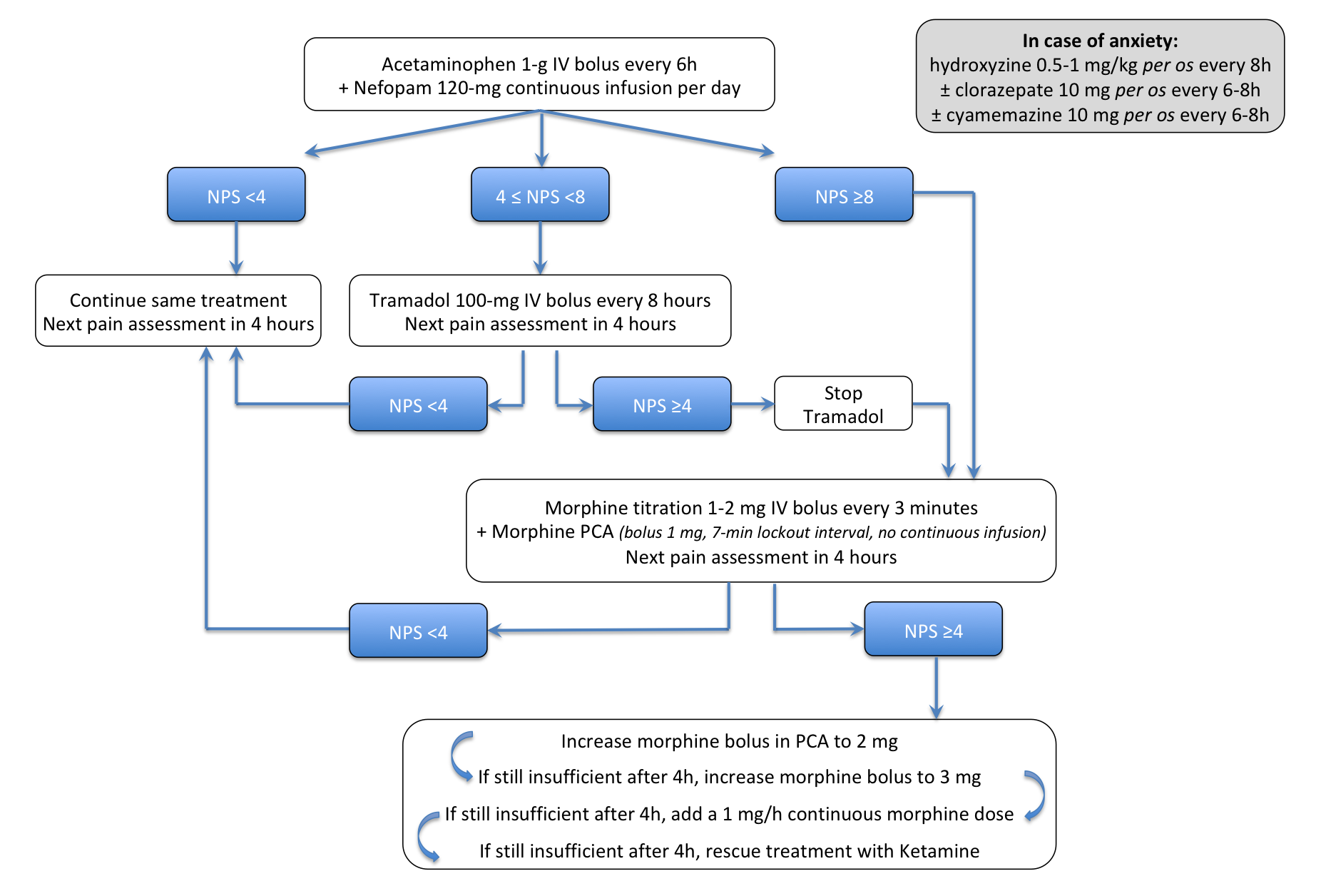
Additional Figure 1. Protocol for pain management of acute chest syndrome in the ICU.**


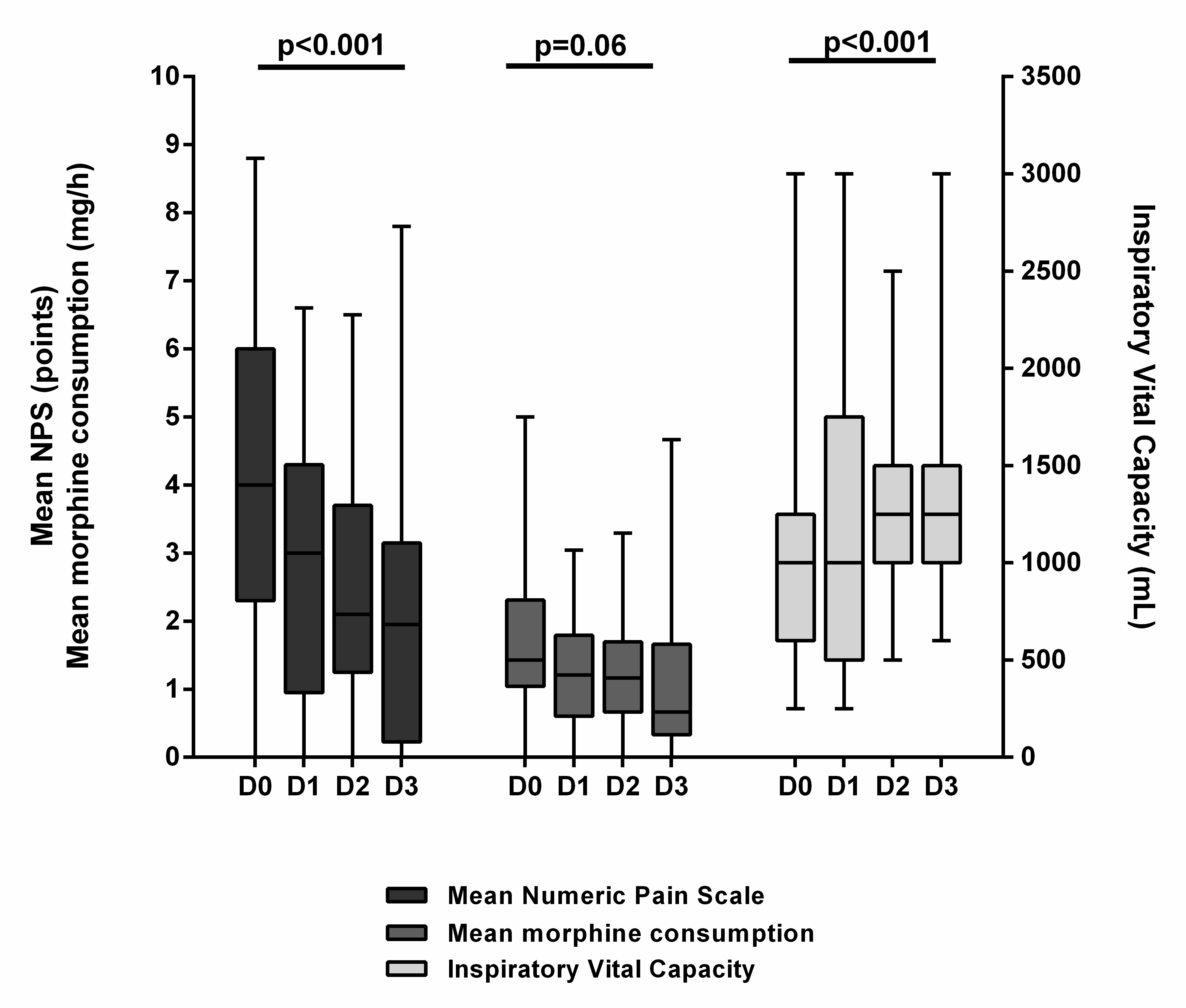


**Additional Figure 2.** **Daily evolution of mean numeric pain scale** (*black boxes, left axis legend*), **morphine consumption** (*dark grey boxes, left axis legend*), **and inspiratory vital capacity** (*light grey boxes, right axis legend*).


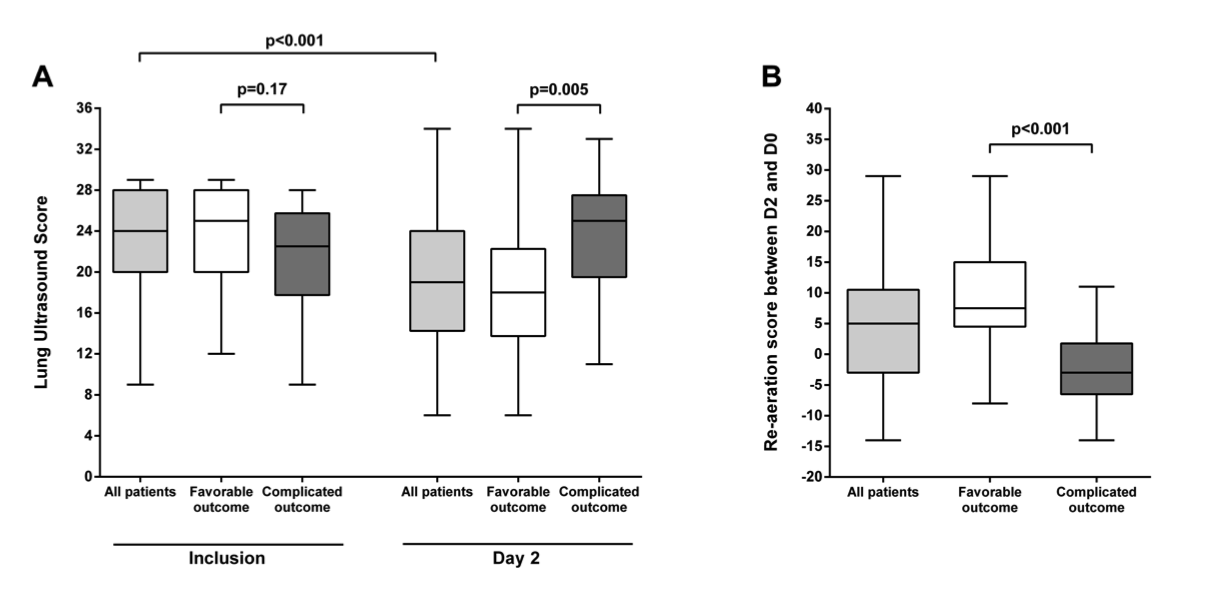


**Additional Figure 3.** **Lung Ultrasound Score (LUS) results (A) at inclusion and 48h after inclusion (day 2), and LU re-aeration score between D2 and D0 (B).** LUS values at inclusion were high (24 [20-28]) (**A**, *left*). Overall LUS values at D2 significantly decreased compared to inclusion (**A**, *right*). Patient with a favourable outcome had a lower LUS-D2 (**A**, *right*) and higher LU re-aeration score (**B**) than patients with a complicated outcome.





**Additional Figure 4. Correlation between Inspiratory Vital Capacity change and the LU re-aeration score between D2 and D0.**


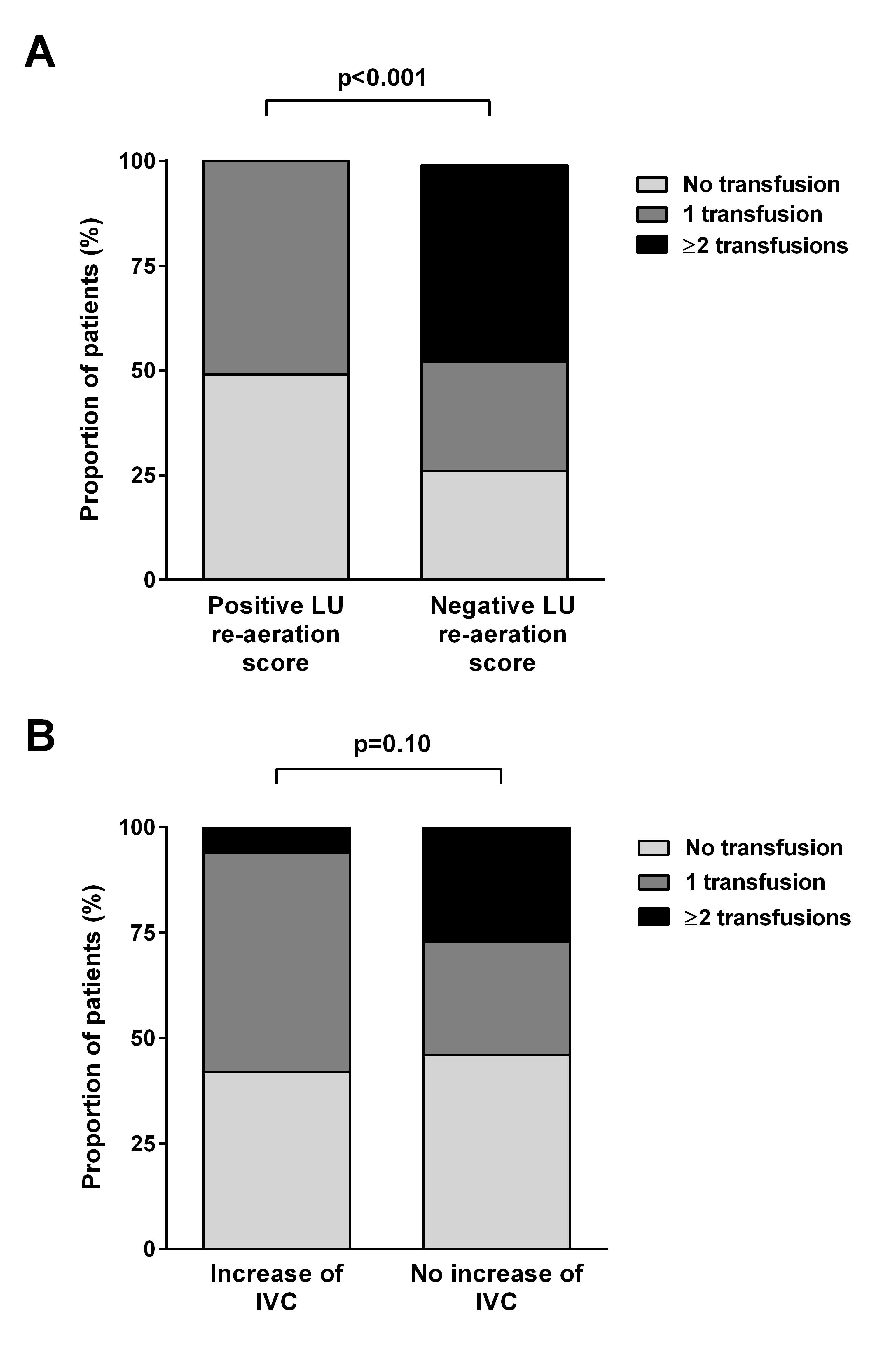
 **Additional Figure 5. Red blood cell transfusions according to the presence or the absence of an improvement of lung aeration assessed by the Lung Ultrasound (LU) re-aeration score or Inspiratory Vital Capacity (IVC).** Patients with a negative LU re-aeration score needed more transfusion episodes than patients with a positive re-aeration score (**A**). Conversely, there was no significant difference regarding the number of transfusion episodes according to IVC change (**B**).
