# Supplementary figures and images for: Morpho-functional evaluation of lung aeration as a marker of sickle-cell acute chest syndrome severity in the ICU: a prospective cohort study
Source: Ann Intensive Care. 2019 Sep 30;9:109. doi: 10.1186/s13613-019-0583-y (PMC6766460; doi:10.1186/s13613-019-0583-y)

## Slide 1
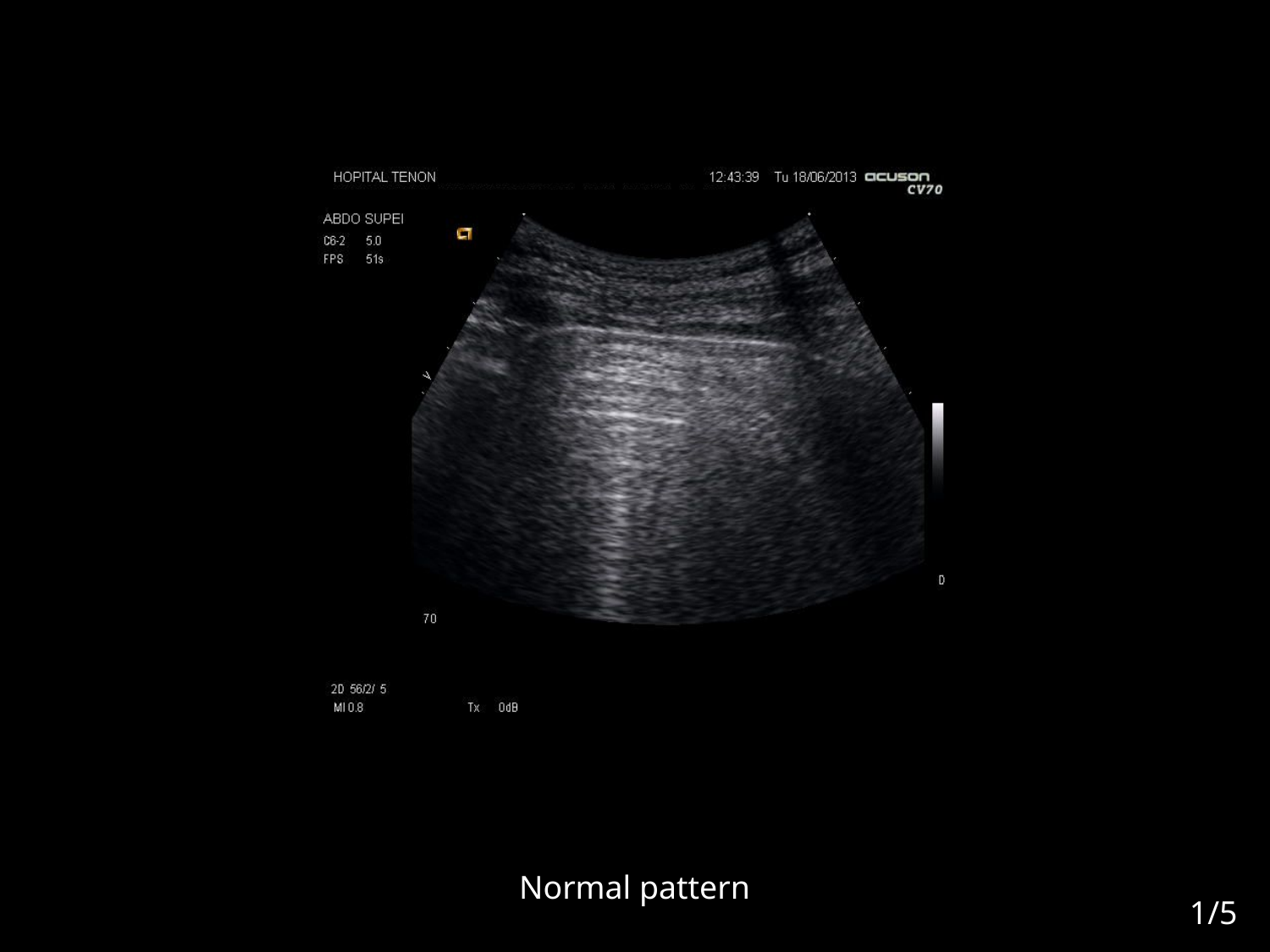

## Slide 2
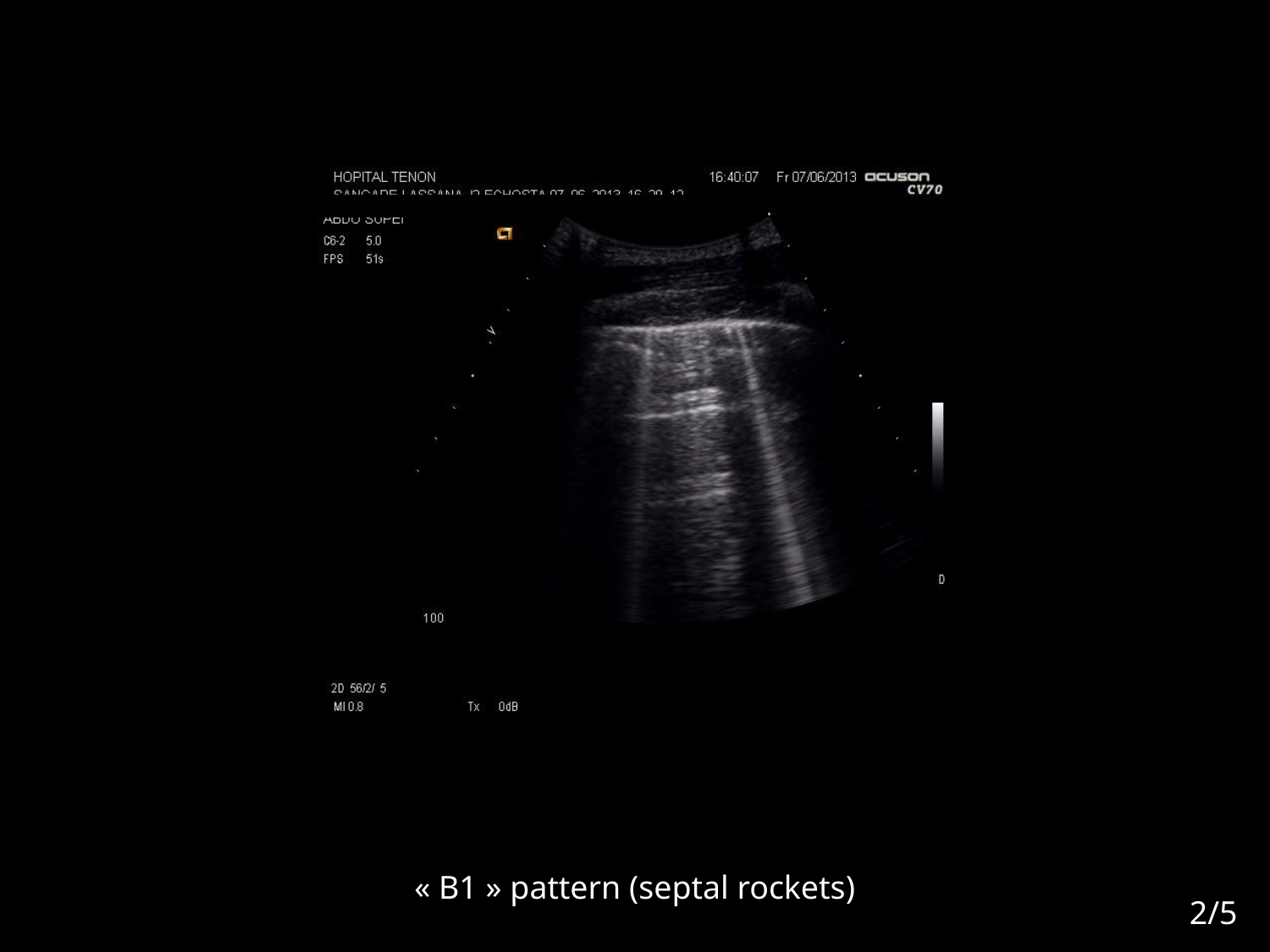

## Slide 3
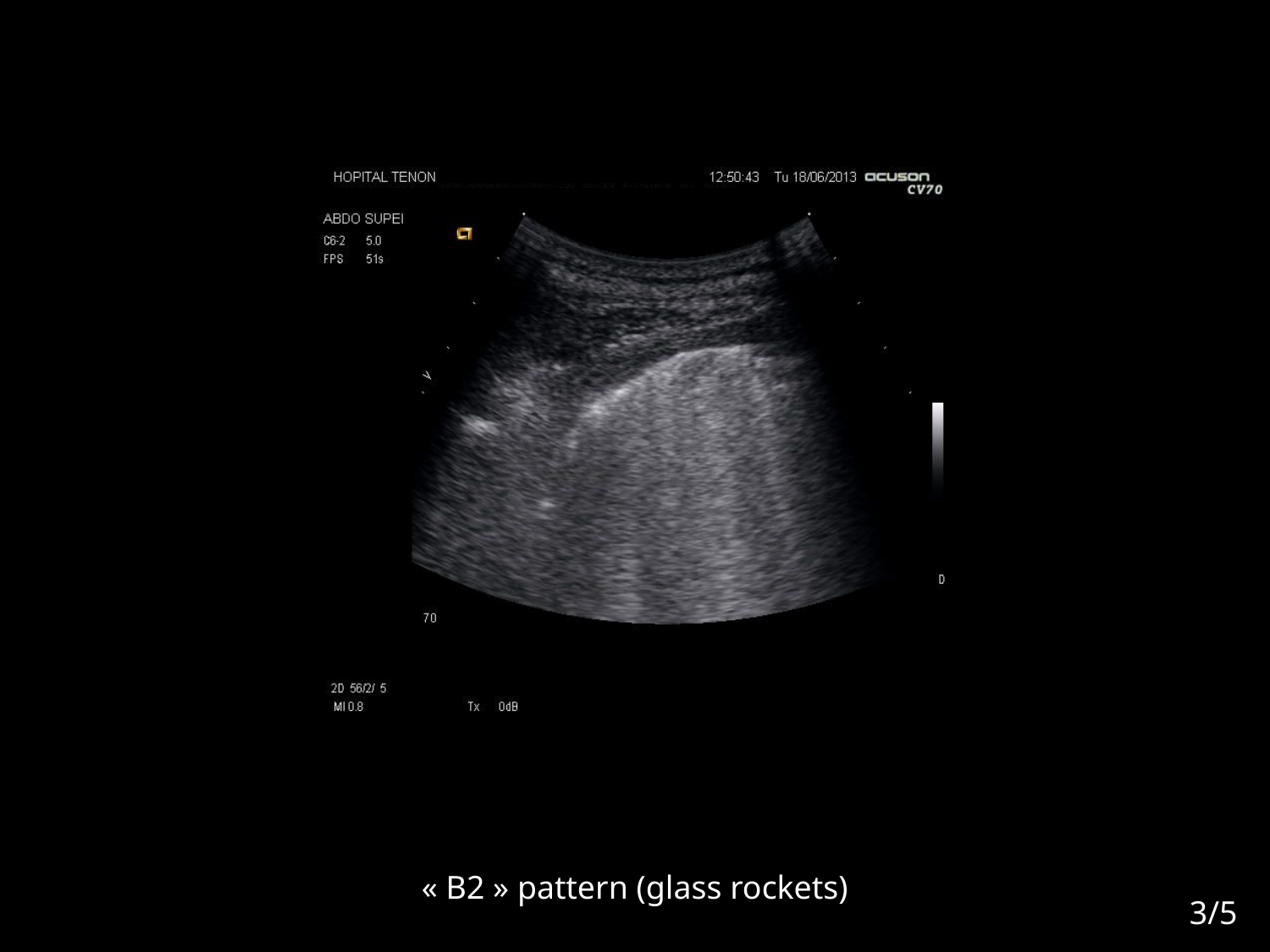

## Slide 4
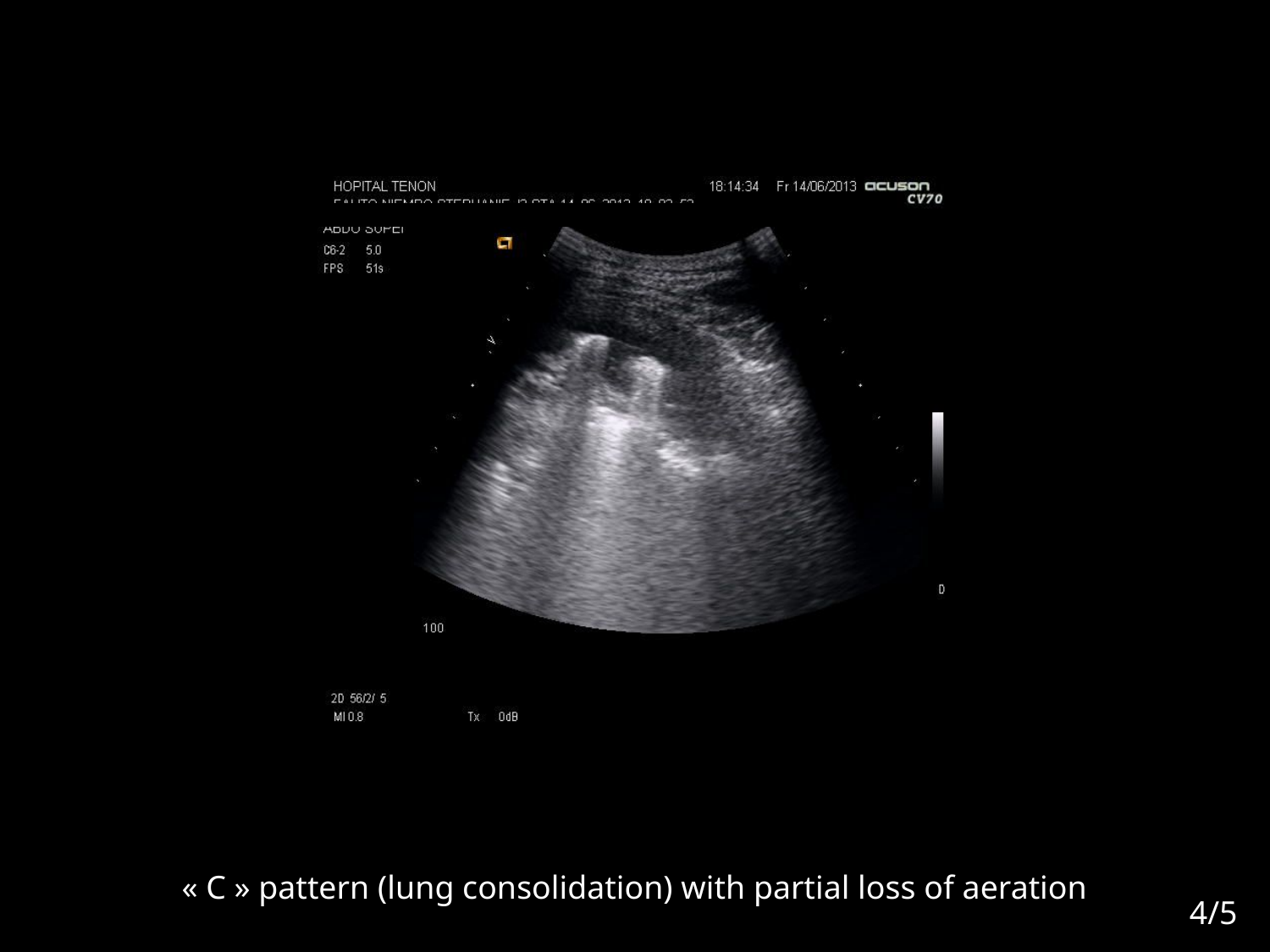

## Slide 5
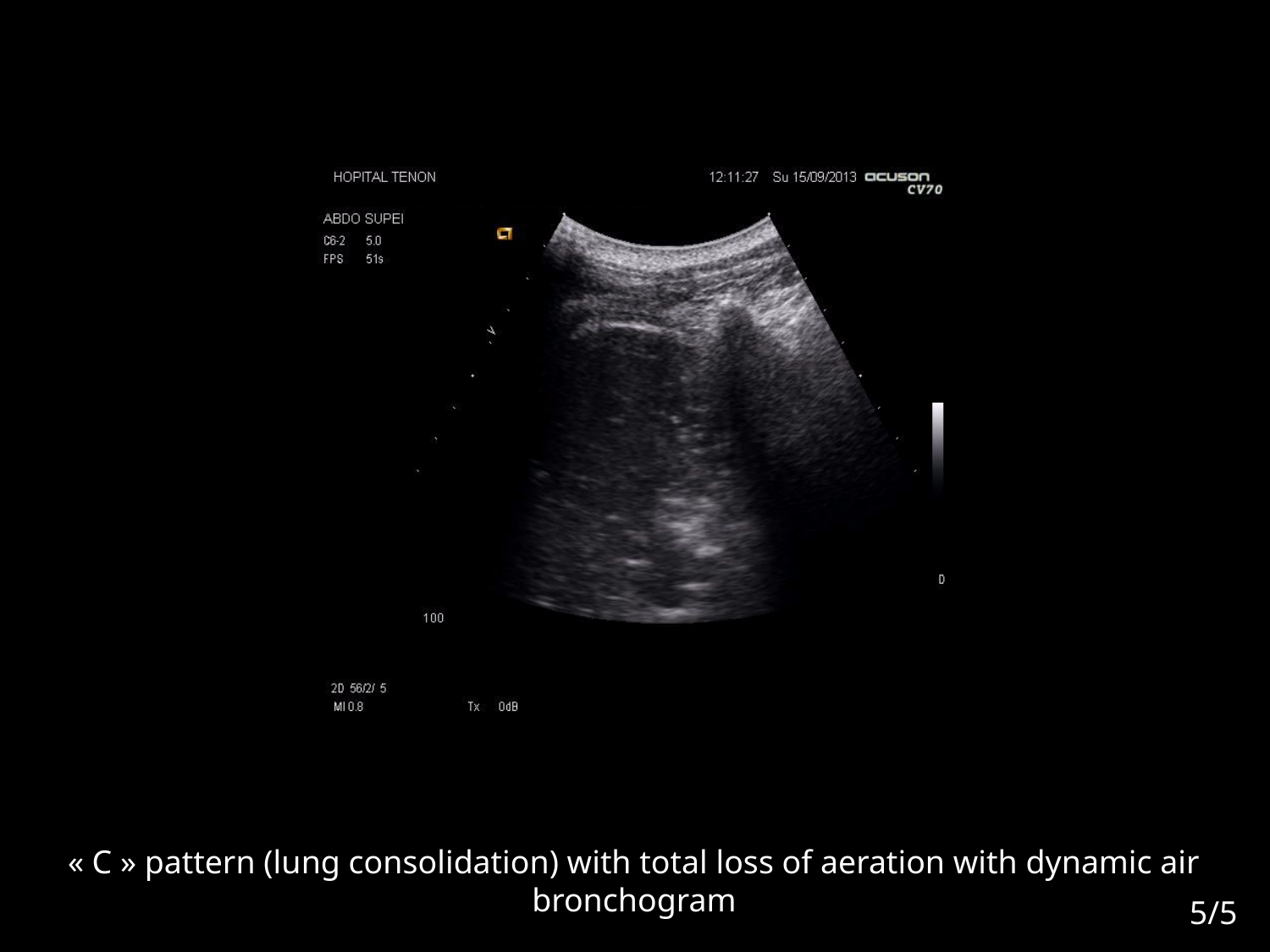

Supplement: Supplementary file 4 — Additional file 4. The additional video file provides LU video loops of the 4 characteristic LU patterns. [file 13613_2019_583_MOESM4_ESM.ppsx]

**Additional Table 1.** Logistic regression of parameters associated with complicated outcome.


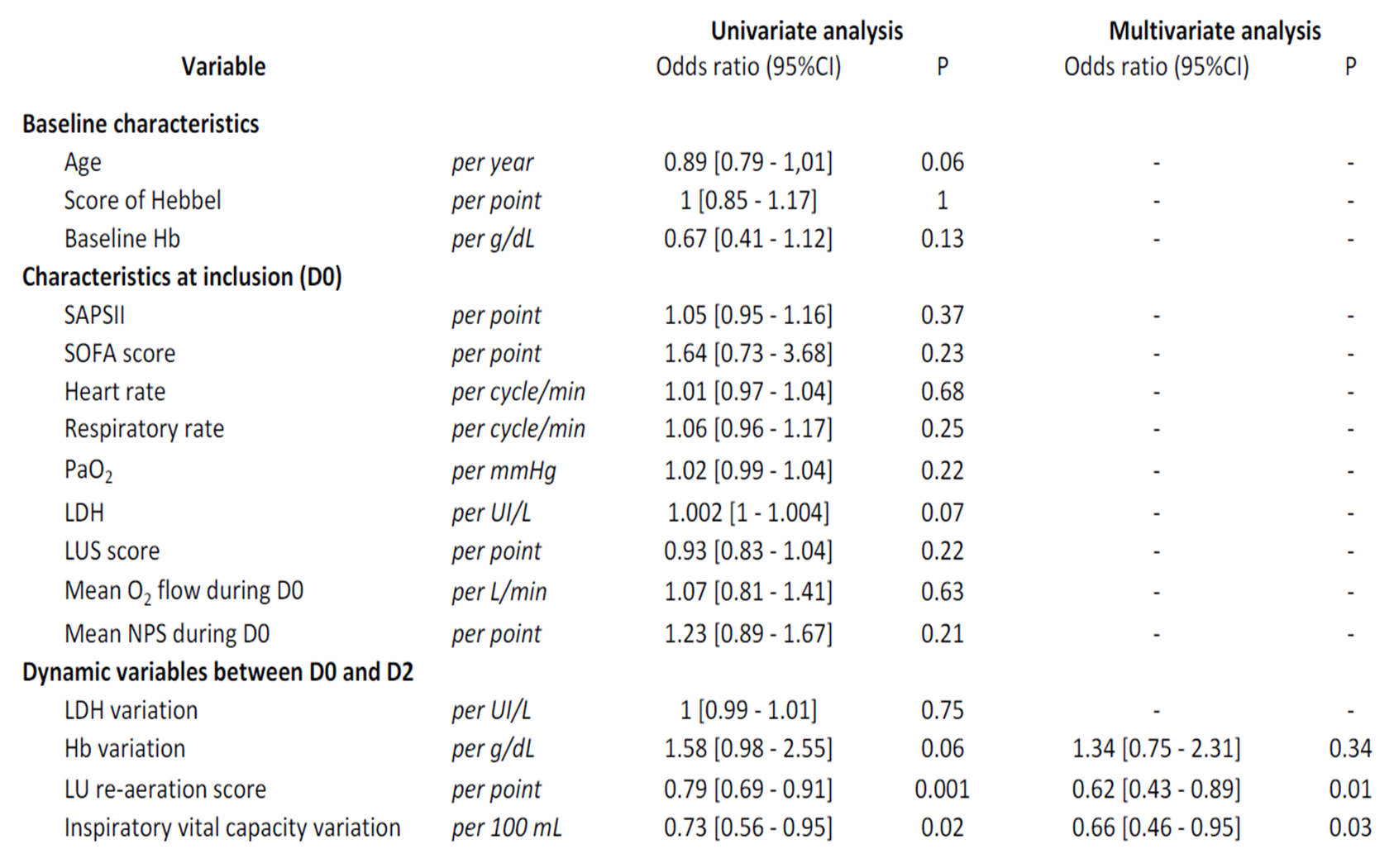

Supplement: Supplementary file 6 — Additional file 6. The additional table file provides the results of the multivariate analysis of parameters associated with complicated outcome. [file 13613_2019_583_MOESM6_ESM.docx]
