# Supplementary material for: Morpho-functional evaluation of lung aeration as a marker of sickle-cell acute chest syndrome severity in the ICU: a prospective cohort study
Source: Ann Intensive Care. 2019 Sep 30;9:109. doi: 10.1186/s13613-019-0583-y (PMC6766460; doi:10.1186/s13613-019-0583-y)
Supplement: Supplementary file 5 — Additional file 5. The additional results file provides additional results on lung ultrasound examination. [file 13613_2019_583_MOESM5_ESM.docx]

**Morpho-functional Evaluation of Lung Aeration as a Marker of Sickle-Cell Acute Chest Syndrome Severity in the ICU: a Prospective Cohort Study**

**Additional Results**

**Lung ultrasound patterns**

Considering the 12 lung regions examined in 56 patients at D0 and in 48 patients at D2 (i.e. 1248 lung regions randomly rated according to the four patterns “N”, “B1”, “B2” and “C”), the κ scores between the 2 LU experts (MG and CA) were 0.94, 0.83, 0.81 and 0.96 for the “N”, “B1”, “B2” and “C” patterns, respectively (global κ score 0.91).

The lung ultrasound patterns for the whole cohort at inclusion are shown in **Figure 2A**. A loss of aeration was found for the majority of patients in all lung regions, except for both left and right antero-superior ones (respectively n°1 and 7). In the latero-inferior and postero-inferior regions, the large majority lung pattern was a lung consolidation, with a height of 4.2 [3-6] and 4.4 [3-5] intercostal spaces for left and right postero-inferior regions, respectively. Forty-three patients (80%) had bilateral postero-inferior lung consolidation. There was no difference in the distribution of lung patterns between patients with favourable and complicated outcomes (p=0.08 - **Figure 2B&C**). The Lung Ultrasound Score (LUS) at inclusion was 24 [20-28], demonstrating the severity of the loss of aeration, without any difference according to patient outcome (**Additional Figure 3A** and **Table 3)**. A static bronchogram was observed in 45 (88%) patients with a left lung consolidation, and 43 (90%) patients with a right lung consolidation. A dynamic bronchogram was observed in 28 (52%) patients with at least one postero-inferior consolidation, while only 6 of them had documented concomitant lung infection (sensitivity 60% and specificity of 37% for the diagnosis of lung infection when a pulmonary consolidation is visualized with LU in an ACS patient). A moderate pleural effusion (<2cm in width) was observed in 14 (25%) and 7 (13%) patients for left and right pleura, respectively.

The lung ultrasound patterns observed at D2 for the 48 patients still hospitalized in the ICU are shown in **Figure 2A.** The loss of aeration regressed moderately, a lung consolidation still involving 79% of patients for the left (n°6) and 69% of patients for the right (n°12) postero-inferior regions. Nevertheless, the height of these postero-inferior lung consolidations significantly decreased from 4.2 [3-6] to 2.9 [1-5] (left) and 4.4 [3-5] to 2.3 [0-4] (right) intercostal spaces (all p<0.001). At D2, the LU pattern was significantly worse in patients with a complicated outcome than in patients with a favourable outcome (p<0.001 - **Figure 2B&C**). Consequently, the LUS at D2 significantly decreased from 25 [20-28] to 18 [14-22] (p<0.001) in patients with a favourable outcome and increased from 22.5 [18-26] to 25 [20-28] in patients with a complicated outcome (**Additional Figure 3A**). The overall LU re-aeration score between D0 and D2 was 5 [-3−9.5]. Fifteen of the 48 patients had a negative re-aeration score, demonstrating a worsening of their loss of lung aeration 48h after ICU admission, of whom 10 had complicated outcome. Moreover, patients with a complicated outcome had a significantly lower re-aeration score than their counterparts (-3 [-6.5–1.8] vs. 7.5 [4.5–15], p<0.001) (**Additional Figure 3B** and **Table 3**). Both the incidences of static (from 90% to 58% of patients) and dynamic (from 52% to 21% of patients) air bronchograms decreased between D0 and D2. A moderate pleural effusion still involved 35% and 19% of patients for left and right pleura, respectively.
